# The Burden of Hypercoagulability in COVID-19

**DOI:** 10.1055/a-1760-0445

**Published:** 2022-02-03

**Authors:** Madeleine Kim, Andrew George, Latha Ganti, Derrick Huang, Matthew Carman

**Affiliations:** 1 The Baylor School, Chatanooga, Tennessee, United States; 2 Department of Biology and Medicine, Brown University, Providence, Rhode Island, United States; 3 Department of Emergency Medicine, University of Central Florida, Orlando, Florida, United States; 4 Department of Emergency Medicine, University of Central Florida College of Medicine, Orlando, Florida, United States; 5 Department of Emergency Medicine, Lakeland Regional Health Medical Center, Lakeland, Florida, United States

**Keywords:** pulmonary embolism, COVID-19, SARS-CoV-2, deep venous thrombosis

## Abstract

The novel coronavirus disease 2019 (COVID-19) infection has widespread impact on multiple organ systems, including damage to endothelial cells. Various studies have found evidence for direct mechanisms by which interaction between severe acute respiratory syndrome-coronavirus-2 (SARS-CoV-2) and endothelial cells lead to extensive damage to the latter, and indirect mechanisms, such as excessively elevated cytokines, can also result in the same outcome. Damage to the endothelium results in release of thrombotic factors and inhibition of fibrinolysis. This confers a significant hypercoagulability burden on patients infected or recovering from COVID-19 infection. In this case report, the authors report the case of a gentleman presenting with extensive deep vein thrombosis and pulmonary embolism, in the context of recent COVID-19 infection. The postulated mechanisms and management are discussed.

## Introduction

The novel coronavirus disease 2019 (COVID-19) was designated as a global pandemic by the World Health Organization on March 11, 2020. COVID-19 is also strongly associated with thrombotic complications, such as deep vein thrombosis (DVT) and pulmonary embolism (PE) which are highly concerning due to associated poorer outcomes. 1 In the emergency department (ED), patients presenting with extremity pain are assessed for risk factors for thrombotic complications, such as a history of DVT, PE, recent surgeries, and active cancer. However, in the setting of COVID-19, patients without traditional risk factors may still be at high risk for thrombosis. The authors present a case of thrombosis in a patient without traditional hypercoagulable risk factors and discuss a review of recent literature on the pathophysiology behind COVID-19 and hypercoagulability.

## Case Presentation

**Fig. 1:**
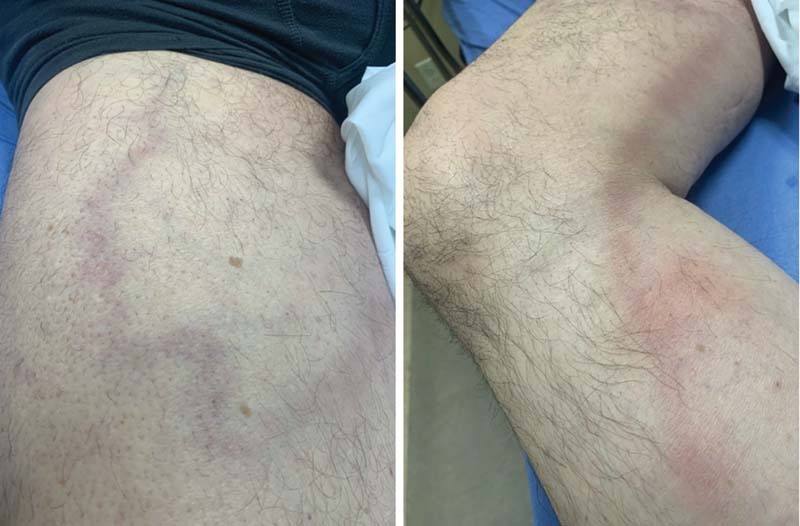
Picture of the patient's leg showing the outline of the painful vein.

A 62-year-old male presented to the emergency department due to right leg pain for 2 days. He elected not to receive the COVID-19 vaccine, and contracted COVID-19 pneumonia 10 days prior, and had been convalescing at home with antibiotics and prednisone. He denied any fevers, chills chest pain, shortness of breath, nausea, vomiting, diarrhea, abdominal pain, headache, or urinary symptoms. The patient had elevated the leg, taken some over-the-counter analgesics, and tried to rest it. However, it had progressively gotten more swollen and painful. The morning of his ED visit, he noticed that the outline of the veins were actually visible, something he had not previously seen ( Fig. 1 ). He also noted that the veins were very hard to touch, in contrast to veins that one can compress. He described the leg as being so painful now that it was hurting even to set it on the floor. The patient had no past medical history besides his COVID-19 illness, and was a nonsmoker. He was also physically quite fit, exercising daily, and working as a martial arts teacher.

On physical examination, his temperature was 98.2°F, pulse 94 beats per minute, respirations 16 breaths per minute, blood pressure 118/80 mm Hg, and his oxygen saturation was 92% on room air. His heart, lungs, abdomen, and neurological status were all unremarkable. Examination of the right leg revealed it to be tender to palpation, mildly edematous, mildly erythematous, and with the outline of the veins clearly visible. The veins were also locally tender to palpation and somewhat hard.

**Fig. 2:**
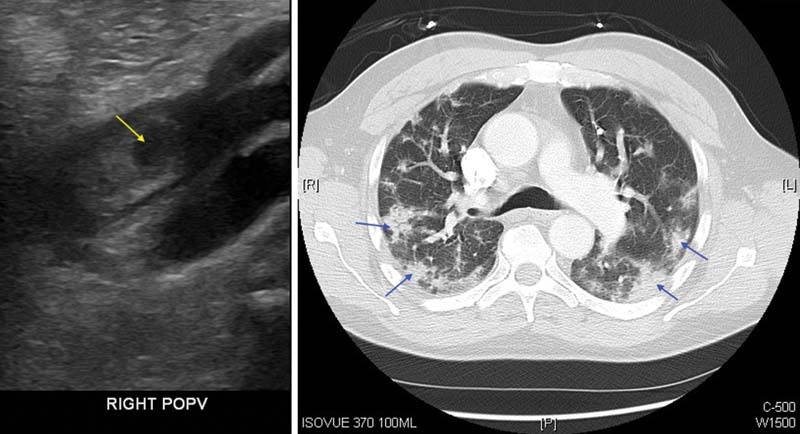
Left panel shows DVT on ultrasonography (yellow arrow); right panel shows CT with ground glass opacities (blue arrows). CT, computed tomography; DVT, deep vein thrombosis.

Duplex venous ultrasonography revealed DVT in the right popliteal and calf veins and superficial vein thrombosis of the greater saphenous vein. Computed tomography angiography scan of the chest revealed left lower lobe pulmonary emboli and extensive bilateral patchy ground-glass airspace opacities ( Fig. 2A and B ). The patient was started on heparin and admitted to the hospitalist service. During hospitalization, his platelet counts rose as high as 562,000 /mm ^3^ , prompting a hematology consult. They performed a myeloproliferative disease workup which was negative. The elevated platelet count was attributed to a reactive thrombosis. The patients was discharged with a direct oral anticoagulant.

## Discussion

Though the mechanisms are not fully known, emerging evidence through recent months regarding the effects of severe acute respiratory syndrome-coronavirus-2 (SARS-CoV-2) infection suggests a state of hypercoagulability associated with COVID-19. 1 Thrombotic complications overall have been a hallmark of COVID-19, with some studies reporting an incidence rate of such complications as high as 79%, even among otherwise healthy, young individuals presenting with the disease. 2 Even more conservative estimates find a significant incidence of adverse thrombotic events in COVID-19 patients, with the absolute best-case scenario rating, the risk at around 9% in non–intensive care unit (ICU) patients (the same study reported a risk of 59% in ICU patients). 3

Common laboratory hallmarks of COVID-19 have been known almost since the initial emergence of the disease to include elevated D-dimers, associated with a poor prognosis. 4 Additional markers include elevated fibrinogen, low platelet counts, and prolonged coagulation times. 5

Recent efforts have resulted in a few different mechanisms being proposed to explain the hypercoagulability state observed in COVID-19 patients. Such explanations seek to provide a link between observed features of COVID-19 and their interaction with the coagulation pathway. Cytokine alterations, endothelial cell injury, platelet activation, neutrophil extracellular traps (NETs), complement activation, and hypoxia all provide, potential explanations, either taken individually or as part of a complex system of interactions and modulation driving the hypercoagulability state.

The first of these comes from the observation of an elevation in proinflammatory cytokines within COVID-19 patients. Of note, key players in the extrinsic coagulation pathway (upstream activators of tissue factor [TF] release) interleukin (IL)-1, IL-6, and tumor necrosis factor (TNF)-α are often elevated in COVID-19 patients. 6 The same cytokines additionally suppress fibrinolysis secondary to increased Plasminogen Activator Inhibitor (PAI)-1 expression. 7 Furthermore, the overall inflammatory state is well known to reduce TF pathway inhibitors, driving coagulation, and potentially working through factor (F)-Xa to enhance inflammation. 8 Finally, such elevated cytokines likely contribute to other purported drivers of hypercoagulability such as NETs and phospholipid responses, discussed in further details shortly.

It is well-reported that COVID-19 can cause damage to endothelial cells. Various studies have found evidence for direct mechanisms by which interaction between SARS-CoV-2 and endothelial cells lead to extensive damage to the latter, and indirect mechanisms, such as excessively elevated cytokines, can also result in the same outcome. 9 10 Damage to the endothelium results in release of thrombotic factors and inhibition of fibrinolysis.

As discussed previously, low platelet counts are a common sign of COVID-19. While the exact reason is unknown (Xu et al proposed three mechanisms: (1) virus-driven decrease in platelet synthesis, (2) virus-driven increased platelet destruction, and (3) virus-driven thrombosis to deplete platelets), 11 the consequence is generally a high degree of platelet activation among remaining platelets due to microthrombi formation, contributing to hypercoagulability. 12 13 Additionally, factors, such as hypoxia common in COVID-19, contribute to platelet activation, along with other hypoxia inducible factor (HIF)-1α mediated prothrombotic and antifibrinolytic effects. 14 15 16

The state of inflammation seen in COVID-19 is also conducive to NETs, used by neutrophils to target pathogens. NETs have been reported to be present in COVID-19 patients, and recent studies have found an association between hypercoagulability and NETs. 17 Indeed, NETs tend to activate the vascular endothelium and platelets to drive thrombosis. 18 Various studies have also linked COVID-19 to elevated complement activation and found associations between elevated complement activation and thrombosis in patients with severe COVID-19. 19 20 Such a role is perhaps unsurprising, given the nature of the complement system and its tendency to drive microthrombi formation, as well as contribute to neutrophil activation and endothelial cell damage, which themselves have been proposed to drive hypercoagulability in COVID-19 patients.

## Conclusion

Ultimately, whether it is one of these mechanisms or something yet to be determined, the clinical outcome of hypercoagulability is nevertheless a serious consideration when treating COVID-19 patients. As observed in this case report, such outcome does not discriminate for health prior to contracting COVID-19.
